# Digitalis Treatment of Pneumonia

**Published:** 1886-10

**Authors:** 


### *DIGITALIS TREATMENT OF PNEUMONIA.*

Petrescu, of the Military Hospital at Bucharest, reports remarkable success in the treatment of acute pneumonia from the administration of large doses of digitalis, given in the form of recent infusion. His conclusions, based upon observation of three hundred and fifty cases, are as follows:

1. Digitalis produces antiphlogistic and direct effects only when given in appropriate doses.

2. This dose is from 4 to 6 grammes (ʒ j–jss) given during the twenty-four hours, and continued for several days. In this way he has given as much as 20 grammes (ʒ v.) inside of three days.

3. The treatment of pneumonia by digitalis is the only method of reducing the mortality of the disease to a minimum.—*Philadelphia Med. Times.*

